# A Modular-Incremental Approach to Improving Compliance Verification With the Biological Weapons Convention

**DOI:** 10.1089/hs.2023.0078

**Published:** 2023-09-25

**Authors:** Nicholas R. Cropper, Shrestha Rath, Ryan J. C. Teo, Kelsey Lane Warmbrod, Mary J. Lancaster

**Affiliations:** Nicholas R. Cropper is a Consultant, Sarasota, FL.; Shrestha Rath, MS, is a Biosecurity Researcher, Effective Ventures Foundation, Oxford, United Kingdom.; Ryan J. C. Teo, MSc, is a Research Assistant, University of Birmingham, Birmingham, United Kingdom.; Kelsey Lane Warmbrod, MPH, MSc, is a PhD Candidate, Institute of Public Health Genetics, University of Washington, Seattle, WA.; Mary J. Lancaster, PhD, is a Senior Scientist, Pacific Northwest National Laboratory, Richland, WA.

**Keywords:** Biological Weapons Convention (BWC), International coordination, Global health diplomacy

## Introduction

The Ninth Review Conference of the Biological Weapons Convention (BWC) concluded in December 2022 with the establishment of a working group that will make recommendations on, among other issues, verification and compliance, enabling discussion on a topic that was sidelined for over 2 decades.^1,2^ Unlike other major disarmament treaties, the BWC does not have an effective mechanism for verifying compliance among states parties. Verification is an essential function of many international disarmament treaties; however, there is little agreement among stakeholders about what form or function verification should take for the BWC.^3^ For the Chemical Weapons Convention^4^ or the Treaty on the Non-Proliferation of Nuclear Weapons,^5^ for example, verification is based on a framework driven by accounting that involves the meticulous documentation of facilities, tools, and raw materials of relevance to the fulfillment of state treaty obligations.^6^

Relying on this accounting approach alone is both technologically and practically infeasible in the context of the BWC.^7^ The dual-use nature of bioscience makes it difficult to differentiate between peaceful and offensive applications from an accounting-driven framework. Furthermore, the increased availability of and ever-widening access to biotechnology has increased the potential for misuse by a variety of nonstate actors. These new, distributed security risks present different verification challenges in the biological context, in contrast with the Chemical Weapons Convention or Treaty on the Non-Proliferation of Nuclear Weapons,^8^ and have led some experts to argue that compliance verification is currently impossible,^3^ although others have pointed out the opportunities that advances in biosciences bring to technical verification.^9^ This commentary offers solutions to some of the political and technical challenges that have led to this conclusion.

No single verification activity can provide unambiguous evidence for assessing treaty compliance. A pragmatic approach to BWC compliance verification should leverage multiple tools and methods to monitor, assess, and evaluate mechanisms to appraise a state party's intent to comply with their obligations to the BWC.^10,11^ The modular-incremental approach proposed in this commentary presents a politically feasible strategy that integrates multiple sources of evidence to provide states parties with contextual information and strengthens opportunities for multilateral consultations, which allow for a collective assessment of BWC compliance.

The lack of a legally enforceable data collecting provision in the BWC treaty contributes to its reliance on politically binding confidence-building measure (CBM) submissions. The CBMs were proposed to facilitate information sharing among states parties regarding biological activities, but the system suffers from low participation—2022 was the first year that over 50% of states parties submitted a CBM.^12,13^ The poor participation rate stems, in part, from the costs and technical difficulties associated with preparing a comprehensive CBM submission, as access to certain information may be distributed across a federal system or may not be collected at all.^12,14^ Moreover, some states do not submit CBMs as they do not necessarily see the benefit from either providing information or receiving it from other states.^15^

## Policy Proposals

### A Modular-Incremental Approach to Implementing a Verification Regime

Previous efforts to build a verification regime for the BWC have been hampered by political and technical challenges.^7,13,16^ To overcome past obstacles to verification, future efforts to develop a verification regime should focus on implementing carefully designed mechanisms that help states parties navigate difficult and ambiguous challenges, such as assessing the intent of a state party to comply with their treaty obligations. This commentary describes a modular-incremental approach, which would achieve this goal by strengthening existing mechanisms and generating more valuable information that would reinforce noncooperation as an indicator of malintent without putting undue suspicion on states parties that are unable to meet their treaty obligations due to technical or resource limitations.

The approach centers around navigating the tradeoff between creating a politically viable and a technically realistic verification mechanism. Three expert-informed, standalone, minimalist improvements to BWC verification mechanisms are described. Each of the *modular* proposals are designed to operate independently and reinforce each other as they are adopted.^17^ These proposals are intentionally designed to be modest and narrow in scope initially in order to achieve *incremental* progress in the face of difficult political and scientific realities.^10^ As states parties become confident in a modest version of each adopted proposal, they can then incrementally expand the scope and authority of each or introduce more modules as they become viable.

Incrementalism is not without challenges. States parties that oppose it have argued that small successes reduce the urgency to negotiate and develop stronger verification measures, in contrast to what they consider the only “sustainable” method of strengthening the BWC, which is to resume negotiations on a comprehensive, legally binding instrument.^18^ However, achieving minor victories might also generate political capital for making progress on more controversial issues.^10^ The pragmatic course of action is to accept that progress toward a functional verification regime is a preferable alternative to waiting for the perfect opportunity to enact a more comprehensive verification protocol.

### Proposal 1: Expand Permanent Institutional Support

Institutional support for the BWC is limited to an understaffed secretariat, known as the Implementation Support Unit (ISU), which is tasked with an overwhelming set of responsibilities.^19,20^ It was only at the recent Ninth Review Conference that the ISU increased its staffing from 3 nonpermanent staff to 4, since its inception in 2006.^1^ Properly supporting a verification mechanism for the BWC will require additional institutional support and a mandate to regularly review CBM submissions and ensure consistency across submissions. Any inconsistencies would be flagged for further review by states parties.^15^

Given the difficulty in achieving consensus for establishing a separate implementing body for the BWC, institutional support for the BWC could begin by focusing on the ISU. The ISU could be strengthened by expanding its mandate to facilitate the resolution of technical disputes between states parties, provide scientific guidance on emerging technologies, and more. One way to go about this expansion, while avoiding over-politicization and maintaining fair geographic representation, is to appoint an expert verification group consisting of a rotating panel of experts overseen by the office of the United Nations Secretary-General. This group would consist of experts ranging from experienced thought leaders specializing in disarmament to industry specialists in emerging biotechnologies serving a fixed term—akin to the World Health Organization's recent call for experts to serve on the Health Security Interface Technical Advisory Group.^21^ As the verification regime is strengthened by the adoption of other proposals, states parties can seek the input of an expanded ISU in strengthening the BWC. Some examples include updating CBMs to align with scientific advancements, establishing shared understandings of data or evidence, or developing and testing field-relevant methods and protocols for data collection.

The role of an expanded ISU could encompass updating states parties on developments in science and technology, providing additional support for Article X,^2^ or developing and testing field-relevant methods and protocols for data collection. However, the first priority for states parties is to agree to establish an international body, such as that proposed by Kazakhstan at a meeting of experts on institutional strengthening of the BWC in 2021,^22,23^ with the mandate to analyze CBM submissions and raise potential noncompliance issues with states parties. CBM submissions can be verified by comparing them with publicly available information gathered through open-source intelligence (OSINT). OSINT refers to intelligence derived from publicly available information, as well as other unclassified information that has limited public distribution or access.^24^ It has been successfully used in conjunction with other sources to monitor compliance with nuclear^25,26^ and chemical weapons^6^ disarmament treaties. Although the dual-use nature of biological activities complicates the types of signals that can be derived from open-source data, the application of OSINT techniques to monitor BWC compliance is not novel.^27,28^ State-level and commercial life science activities can also be subject to public monitoring, including through the surveillance of publications and legislation.^29^ The ever-growing digitalization of commercial and academic research activities is generating a greater volume of data that may be useful for assessing states' compliance with the BWC.

In addition to confirming the accuracy of CBM submissions, OSINT could help the expert verification group uncover additional facts during future disputes, create previously overlooked links between seemingly unrelated sources, and generate a more complete picture of any data collected to support verification activities. This information is essential for assessing intent to comply with treaty obligations. If OSINT produces factual findings that contradict a state party's CBM submissions or public statements, that information may warrant further consultation to either reassure other states parties of their compliance or uncover further evidence of noncompliance.

### Proposal 2: Universalize Confidence-Building Measure Submissions

The original intention behind the current system of CBM submissions was to improve transparency and confidence among states parties.^30^ However, the abysmally low submission rate for CBMs defeats the purpose of reducing doubts and ambiguities in the field of peaceful biological activities.^7,12^ This failing signals either an unwillingness to contribute, potentially indicating disinterest or even malintent, or an inability to contribute to the system due to logistical and resource challenges.^12,14,31^ Overcoming this obstacle requires refocusing on collecting information most relevant to verifying compliance, incentivizing CBM submissions, and reducing the technical barriers to gathering relevant information.

To maximize the informational value of CBMs, submissions should incrementally be made mandatory on a form-by-form basis while offsetting the burden on states lacking technical expertise. Form E, which requires states parties to declare legislation and regulations related to national implementation, is an ideal starting point for mandating CBM submissions because it reveals how much effort well-resourced states parties are putting into national implementation.^14^ Form E also would be useful for identifying states parties that may require support for national implementation, optimizing the distribution of technical and logistical assistance under any Article X mutual assistance programs.

Current efforts to strengthen national implementation of CBMs through training workshops are usually subject to the availability of funding by individual states parties.^32,33^ To ensure that less technically capable states parties are able to meet new proposed treaty obligations, Article X should be enhanced by requiring that financial resources and technical expertise be shared among states parties through a system similar to existing benefit sharing systems, such as the Pandemic Influenza Preparedness Framework.^34^ Pairing mandatory CBM submissions with additional assistance under Article X would ease the burden of mandating CBM submissions and incentivize voluntary submissions.^13^

Apart from sharing technical expertise through an enhanced mutual assistance program, states parties may also be encouraged to submit CBMs if doing so were made logistically easier. The process of preparing CBM submissions involves compiling and sharing a large volume of data drawn from a variety of sources and is complicated by the lack of a consistent reporting standard.

Recent advances in artificial intelligence and natural language processing have the potential to make some CBM submissions easier, such as through the use of data harmonization and text mining. Data harmonization refers to the process of integrating and standardizing heterogeneous data structures to ensure consistency and compatibility.^35^ This has been successfully applied to analyze multisource data trends in fields ranging from banking^36^ to healthcare^37^ and could also simplify data collection for use in CBM submissions. Furthermore, text mining, which uses natural language processing to extract information from large amounts of unstructured text data,^38^ can be used to complement human judgment in the compilation and analysis of CBM submissions, which would otherwise be tedious. These applications are illustrated using large language models to successfully conduct structured information extraction on complex scientific text, although it should be accompanied by human oversight.^39^

Text mining and data harmonization are useful not only for states parties, but they can also be leveraged by the proposed expert verification group in 2 ways. First, these technologies can dramatically reduce the effort required to analyze CBM submissions and accelerate their contribution to investigative mechanisms. Second, text mining can be augmented by automation to enable continuous, passive CBM monitoring. This can help uncover suspicious inconsistencies or activities in CBM submissions that might otherwise be overlooked by manual analysis.^27,40^

These new tools will not replace human review, nor will they eliminate all the technical challenges many states parties face when preparing CBM submissions. Rather, text mining and data harmonization can simplify data analysis and facilitate reporting generalized data not targeted at any specific state party. If properly implemented, these tools could contribute to greater transparency in biological activities globally and increase the informational value of CBMs to states parties.

### Proposal 3: Improve the Collaborative Investigative Mechanism Under Article V

Currently, Article VI provides for a legally binding mechanism to investigate allegations of noncompliance with the BWC.^2,41^ This was triggered for the first time in 2022 by the Russian Federation against Ukraine and the United States.^42^ In line with expectations, politics within the United Nations Security Council blocked any attempt to initiate an Article VI investigation.^43^ Going forward, some states parties may turn to the United Nations Secretary-General's Mechanism for Investigation of Alleged Use of Chemical and Biological Weapons (UNSGM),^44^ but it is not an ideal investigatory mechanism as it is designed to respond only to alleged use of chemical and biological weapons and would therefore have to be activated *after* the deployment of a chemical and biological weapon.^45^ It is critical for the scope of investigative mechanisms to include allegations of other forms of noncompliance, including the development, production, and stockpiling of weapons.

An alternative investigative mechanism is needed to cooperatively review evidence and investigate potential noncompliance. Through Article V, states parties developed a formal consultative meeting that has proven procedurally successful in the past as a forum for states parties to present evidence in an open setting. However, the Article V mechanism is confrontational and prone to political abuse, as past consultations have historically been used to question whether states parties are acting in good faith or actively violating the BWC.^46^ This mechanism could be greatly improved by the creation of a third-party review process with 2 goals: preventing baseless accusations from becoming political stunts and making the activation of the current Article V mechanism less accusatory. There is precedence for various approaches to this idea in the Aarhus Convention,^47^ the International Health Regulations,^48,49^ and the Chemical Weapons Convention.^50^ One such approach is to have requests be submitted to a politically neutral chairperson or a rotating group of states parties for review. With the consultation of experts, potentially from the expanded ISU, the third party could dismiss requests or proceed with a formal consultative meeting where evidence can be presented and evaluated by all states parties.

These proposals will likely not eliminate the political challenges that a voluntary consultative mechanism faces in a complex geopolitical climate. However, this proposed improvement to Article V need not preclude or undermine the option of seeking an Article VI or UNSGM investigation. Rather, the goal is to establish a system for transparently, collaboratively, and scientifically assessing accusations while maintaining the threat of a more “forceful” approach through the UNSGM or Article VI. Furthermore, with the advancement of new tools to detect^51^ and, eventually, attribute genetically engineered organisms and DNA sequences, the ever-improving quality of evidence in support of noncompliance will reduce ambiguities in the investigative procedure. Genetic engineering detection and attribution tools cannot entirely overcome the political challenge of assigning blame but can serve to reduce the likelihood of investigations becoming politicized.

### Funding

Any verification mechanism will require additional funding for the BWC. Similarly ambitious disarmament treaties receive budgets many times that of the BWC.^52,53^ Inconsistent financial resources have stymied work to modernize the BWC, including shortened or canceled intersessional meetings, stalled implementation of decisions, limited institutional support, and little direct assistance for national implementation.^12,14,54^ Additional funding, perhaps sourced from an agreement on expanding the system of mandatory dues or from a permanent budget provided by another global governance body, is critical for developing a workable verification regime.

## Conclusion

Establishing a verification mechanism for the BWC is a politically and technically complex challenge that cannot be solved by any single tool or implementation scheme. Relying on verification approaches that were successful for chemical and nuclear weapons disarmament treaties are unlikely to succeed for the BWC. This is because any regime must acknowledge the ambiguities created by 21st century biotechnologies and the challenge of applying material-focused verification regimes to biological weapons. The modular-incremental approach is a flexible strategy that provides guidance to states parties for establishing the pillars of a scientifically robust verification regime without overprescribing specific remedies that may not fit their capacity or circumstances. When states parties find themselves unable to progress on a particular module, they could pivot to considering other modules, incrementally enhancing existing mechanisms, or applying novel scientific tools to existing data or mechanisms. The incremental approach presents an opportunity to increase the credibility of these technologies among states parties as each validated application increases confidence that an extension could be successful. Every minor success generates political capital to spend on overcoming other barriers, and previously stalled ideas can be revisited with new technologies and personalities in the future.

Our proposals grapple with the fundamental challenge of evaluating intent and the difficult ambiguities arising from the dual-use nature of modern bioscience. By developing mechanisms enabled by modern scientific tools, our work tries to eliminate opportunities for would-be violators to hide behind ambiguity. Data harmonization and mutual assistance programs lower the barrier to CBM submissions, leaving little reason for failure other than purposeful circumvention. OSINT and institutional data reviews can raise red flags. Enhancing Article V creates a forum for collaboratively reviewing evidence, like that obtained from genetic engineering detection, OSINT, and relevant CBMs, to better assess responsible applications of biology and what the user's motivations might be.

Despite a strong norm against the use of biological weapons, the lack of a verification mechanism severely weakens the BWC. Bioscience, during the past 2 decades of stalled negotiations, has seen revolutionary advancements that have radically redefined what is possible, for better and for worse. In this context, the inability to govern the use of biological weapons effectively could have potentially catastrophic consequences. Incrementalism has the potential to break the political logjam and help governments keep up with rapid innovations in the biosciences. The pace of incrementalism is not ideal, but continued inaction is unacceptable.
